# Obesity and Outcomes of Kawasaki Disease and COVID-19–Related Multisystem Inflammatory Syndrome in Children

**DOI:** 10.1001/jamanetworkopen.2023.46829

**Published:** 2023-12-08

**Authors:** Michael Khoury, Ashraf S. Harahsheh, Geetha Raghuveer, Nagib Dahdah, Simon Lee, Marianna Fabi, Elif Seda Selamet Tierney, Michael A. Portman, Nadine F. Choueiter, Matthew Elias, Deepika Thacker, Frédéric Dallaire, William B. Orr, Tyler H. Harris, Kambiz Norozi, Dongngan T. Truong, Manaswitha Khare, Jacqueline R. Szmuszkovicz, Joseph J. Pagano, Cedric Manlhiot, Pedrom Farid, Brian W. McCrindle

**Affiliations:** 1 Division of Pediatric Cardiology, Department of Pediatrics, University of Alberta, Edmonton, Alberta, Canada; 2 Children’s National Hospital, The George Washington University School of Medicine & Health Sciences, Washington, DC; 3 Children’s Mercy Hospital, Kansas City, Missouri; 4 Division of Pediatric Cardiology, CHU Ste-Justine, University of Montreal, Montreal, Quebec, Canada; 5 The Heart Center at Nationwide Children’s Hospital, Columbus, Ohio; 6 Pediatric Emergency Unit, IRCCS Azienda Ospedaliero Universitaria di Bologna, Bologna, Italy; 7 Lucile Packard Children’s Hospital, Stanford University Medical Center, Palo Alto, California; 8 Seattle Children’s Research Institute, Seattle, Washington; 9 Children’s Hospital at Montefiore, Albert Einstein College of Medicine, Bronx, New York; 10 Children’s Hospital of Philadelphia, Philadelphia, Pennsylvania; 11 Nemours Children’s Hospital, Wilmington, Delaware; 12 Department of Pediatrics, Universite de Sherbrooke, and Centre de Recherche du Centre Hospitalier Universitaire de Sherbrooke, Sherbrooke, Quebec, Canada; 13 Division of Pediatric Cardiology, Department of Pediatrics, Washington University School of Medicine, St Louis, Missouri; 14 UPMC Children’s Hospital of Pittsburgh, Pittsburgh, Pennsylvania; 15 Department of Pediatrics, Pediatric Cardiology, Western University, London, Ontario, Canada; 16 University of Utah and Primary Children’s Hospital, Salt Lake City; 17 University of California San Diego/Rady Children’s Hospital San Diego; 18 Children’s Hospital of Los Angeles, Los Angeles, California; 19 Blalock-Taussig-Thomas Congenital Heart Center at Johns Hopkins University, Baltimore, Maryland; 20 Labatt Family Heart Centre, The Hospital for Sick Children, Department of Pediatrics, University of Toronto, Toronto, Ontario, Canada

## Abstract

**Importance:**

Obesity may affect the clinical course of Kawasaki disease (KD) in children and multisystem inflammatory syndrome in children (MIS-C) associated with COVID-19.

**Objective:**

To compare the prevalence of obesity and associations with clinical outcomes in patients with KD or MIS-C.

**Design, Setting, and Participants:**

In this cohort study, analysis of International Kawasaki Disease Registry (IKDR) data on contemporaneous patients was conducted between January 1, 2020, and July 31, 2022 (42 sites, 8 countries). Patients with MIS-C (defined by Centers for Disease Control and Prevention criteria) and patients with KD (defined by American Heart Association criteria) were included. Patients with KD who had evidence of a recent COVID-19 infection or missing or unknown COVID-19 status were excluded.

**Main Outcomes and Measures:**

Patient demographic characteristics, clinical features, disease course, and outcome variables were collected from the IKDR data set. Using body mass index (BMI)/weight *z* score percentile equivalents, patient weight was categorized as normal weight (BMI <85th percentile), overweight (BMI ≥85th to <95th percentile), and obese (BMI ≥95th percentile). The association between adiposity category and clinical features and outcomes was determined separately for KD and MIS-C patient groups.

**Results:**

Of 1767 children, 338 with KD (median age, 2.5 [IQR, 1.2-5.0] years; 60.4% male) and 1429 with MIS-C (median age, 8.7 [IQR, 5.3-12.4] years; 61.4% male) were contemporaneously included in the study. For patients with MIS-C vs KD, the prevalence of overweight (17.1% vs 11.5%) and obesity (23.7% vs 11.5%) was significantly higher (*P* < .001), with significantly higher adiposity *z* scores, even after adjustment for age, sex, and race and ethnicity. For patients with KD, apart from intensive care unit admission rate, adiposity category was not associated with laboratory test features or outcomes. For patients with MIS-C, higher adiposity category was associated with worse laboratory test values and outcomes, including a greater likelihood of shock, intensive care unit admission and inotrope requirement, and increased inflammatory markers, creatinine levels, and alanine aminotransferase levels. Adiposity category was not associated with coronary artery abnormalities for either MIS-C or KD.

**Conclusions and Relevance:**

In this international cohort study, obesity was more prevalent for patients with MIS-C vs KD, and associated with more severe presentation, laboratory test features, and outcomes. These findings suggest that obesity as a comorbid factor should be considered at the clinical presentation in children with MIS-C.

## Key Points

**Question:**

What is the prevalence of obesity and association with clinical presentation and outcomes in children with Kawasaki disease (KD) and multisystem inflammatory syndrome in children (MIS-C)?

**Findings:**

In this cohort study including 1767 patients with KD or MIS-C, the prevalence of overweight and obesity was significantly higher in patients with MIS-C compared with KD (41% vs 23%). Obesity was not associated with most clinical presentations or outcomes in patients with KD, but was associated with a more severe presentation and worsened outcomes in patients with MIS-C.

**Meaning:**

The findings of this study suggest that obesity is a comorbid factor that should be considered at the clinical presentation in children with MIS-C.

## Teaser

This cohort study examines the prevalence of obesity and its association with clinical outcomes in children with Kawasaki disease and multisystem inflammatory syndrome in children (MIS-C) associated with COVID-19.

## Introduction

While children have generally been more predisposed toward asymptomatic or mild disease with acute SARS-CoV-2 (COVID-19) infections, a more serious postinfectious inflammatory syndrome was identified early in the pandemic, termed *multisystem inflammatory syndrome in children* (MIS-C).^1,2^ The overlap of cardiac and extracardiac manifestations has inextricably linked MIS-C and Kawasaki disease (KD), a medium-vessel vasculitis in childhood of uncertain etiology that remains a leading cause of pediatric-acquired heart disease worldwide, especially in high-income countries.^3^ The rapidity and severity by which MIS-C emerged as a post–COVID-19 acute complication left clinicians to develop treatment strategies often in the absence of evidence. Thus, clinicians relied on the incorporation of treatments used for similar disease processes, such as KD.^4^

Despite their similarities, distinct differences exist between MIS-C and KD. For example, while the primary cardiac consequence of concern with KD is the development of coronary artery aneurysms,^3^ patients with MIS-C have a propensity toward cardiogenic shocklike presentations with inotropic requirements and myocardial dysfunction.^5,6^ Due to the severity of clinical presentation, there has been a strong interest among researchers to identify any associations between various demographic, clinical, and laboratory factors with outcomes in MIS-C. Specifically, obesity has been evaluated in multiple studies, in part due to its known role as a risk factor associated with hospitalization, intensive care unit (ICU) admission, and death in children with acute COVID-19 infection.^6,7,8,9,10,11,12,13,14^ Associations between obesity and MIS-C outcomes, however, have been found inconsistently across studies to date.^6,12,15,16,17^ Moreover, studies evaluating the outcomes of children with obesity and KD remain largely lacking, apart from a single study.^18^ Given that excessive adipose tissue in individuals with obesity is associated with a systemic inflammatory state,^19^ it is possible that obesity mediates disease severity in both KD and MIS-C. Thus, we sought to compare the prevalence of obesity in patients with KD and in patients with MIS-C and examine whether there are associations between obesity and clinical outcomes for these patients.

### Methods

Data for this study were obtained from the International Kawasaki Disease Registry (IKDR) between January 1, 2020, and July 31, 2022. This study and report followed the Strengthening the Reporting of Observational Studies in Epidemiology (STROBE) reporting guideline for cohort studies.^20^ The IKDR enrolled contemporaneous patients (as of January 2020) with acute KD and MIS-C, and patients with cardiac complications secondary to acute COVID-19 from 42 sites and 8 countries.^21^ Data were abstracted from medical records at each participating site, including patient demographic characteristics, clinical features, disease course (including COVID-19 testing status, serial laboratory test values, and cardiac imaging reports), as well as management and clinical outcomes (including admission to the ICU, medications used, and death). Data on race and ethnicity, where available, were collected from site electronic medical records. Depending on the study site, race and ethnicity were either entered routinely for clinical purposes or for research reporting. All data were entered into a secure REDCap database^22^ maintained by the IKDR Data Coordinating Center (DCC), located at The Hospital for Sick Children, Toronto, Canada. On review by the DCC, queries were sent to sites for clarifications and revisions as needed, requiring all queries to be resolved before patient inclusion in the data analysis. All participating sites maintain institutional review board approval and have data-sharing agreements with the DCC. All patients and parents provided written informed consent or assent, or data were submitted with an approved waiver of consent (as per the local institutional review board’s requirement). Patient identifiers were removed from all data submitted to the DCC.

Multisystem inflammatory syndrome in children was diagnosed by each site and confirmed by the DCC to meet the Centers for Disease Control and Prevention criteria, including the requirement for documented evidence of prior COVID-19 infection.^23^ Kawasaki disease was diagnosed by each site and confirmed by the DCC to meet the American Heart Association guideline criteria (both patients with complete and incomplete KD were included).^3^ Given the major phenotypic overlap possible between MIS-C and KD, KD cases in the setting of documented evidence of a prior COVID-19 infection (or missing or unknown COVID-19 status) were excluded from analysis. Patients were also excluded if their height and weight were not recorded. The degree of adiposity was determined by using the World Health Organization weight normative values for patients younger than 2 years^24^ and the Centers for Disease Control and Prevention body mass index (BMI; calculated as weight in kilograms divided by height in meters squared) for those aged 2 years or older.^25^ This was initially expressed as *z* score (which we refer to as adiposity *z* score), and then converted to percentiles. Adiposity categories were defined as overweight (BMI/weight ≥85th to <95th percentile), obesity (BMI/weight ≥95th percentile), and severe obesity (BMI/weight >99th percentile).^25^

### Statistical Analysis

Data are described as frequencies, medians with IQRs, and means with SDs, as appropriate. To compare demographic and clinical characteristics of patients with KD vs MIS-C, χ^2^ tests, *t* tests, and Kruskal-Wallis analysis of variance were used as appropriate. To determine differences in diagnosis related to markers of adiposity, groups were compared for adiposity *z* score (general linear regression modeling) and ordinal adiposity category (Kruskal-Wallis analysis of variance). Furthermore, group comparison for adiposity *z* score was adjusted for age, sex, and race and ethnicity. As associations were likely to be nonlinear, ordinal adiposity category based on percentile was used (normal weight, overweight, and obese). To determine the association of ordinal adiposity category with demographic and clinical characteristics, Mantel-Haenszel χ^2^ and Kruskal-Wallis analysis of variance tests were used, stratified by diagnosis group. Statistical significance was defined as *P* < .05. Statistical analysis was performed using SAS version 9.4 (SAS Institute Inc).

## Results

**Figure 1.**
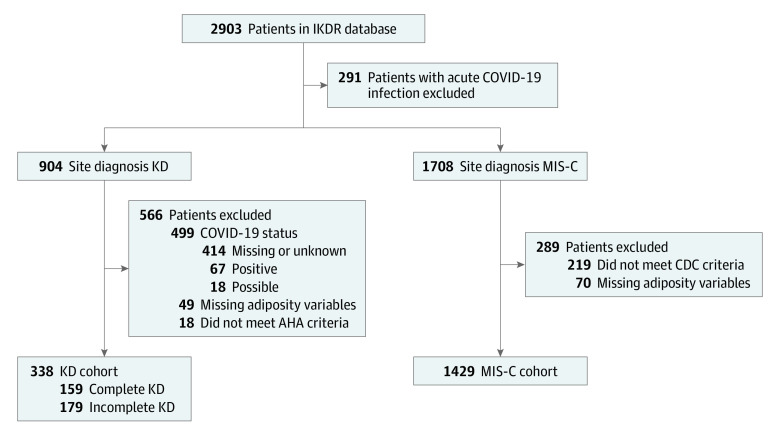
Flowchart Describing the Study Cohort AHA indicates American Heart Association; CDC, Centers for Disease Control and Prevention; IKDR, International Kawasaki Disease Registry; KD, Kawasaki disease; and MIS-C, multisystem inflammatory syndrome in children.

There were 2903 patients in the IKDR database during the study period. After exclusion of patients with acute COVID-19 infection; patients not meeting diagnostic criteria for KD or MIS-C; patients with KD who had missing or unknown, possible, or positive COVID-19 status; and patients with missing adiposity status, 1767 patients remained (338 with KD; 60.4% male; 39.6% female; median age, 2.5 [IQR, 1.2-5.0] years and 1429 with MIS-C; 61.4% male; 38.6% female; median age, 8.7 [IQR, 5.2-12.4] years) (Figure 1, Table 1). Compared with patients with KD, those with MIS-C were older (*P* < .001), with a similar proportion of males (*P* = .72). Patient data were included from 8 countries, with 89.7% coming from the US or Canada.

**Table 1.** Descriptive Characteristics of Patients With Kawasaki Disease and MIS-C

Characteristic	Kawasaki disease (n = 338)		MIS-C (n = 1429)		*P* value
	No.	No. (%)	No.	No. (%)	
Sex (% males)	338		1429		
Male		204 (60.4)		878 (61.4)	.72
Female		134 (39.6)		551 (38.6)	
Age, median (IQR), y	338	2.5 (1.2-5.0)	1429	8.7 (5.3-12.4)	<.001
Race and ethnicity	233		982		
Black		35 (15.0)		270 (27.5)	<.001
East Asian		27 (11.6)		7 (0.7)	<.001
Hispanic		49 (21.0)		283 (28.8)	.02
South Asian		13 (5.6)		66 (6.7)	.53
White		93 (39.9)		308 (31.4)	.02
Other		16 (6.9)		48 (4.9)	.23
Shock at presentation	332	9 (2.7)	1426	487 (34.2)	<.001
ICU admission	335	22 (6.6)	1428	815 (57.1)	<.001
Received inotropic agents	338	27 (8.0)	1428	648 (45.4)	<.001
Death	334	0	1353	8 (0.6)	.37
Hospital length of stay, median (IQR), d	338	5 (4-7)	1429	7 (5-10)	<.001
Worst LVEF, mean (SD), %	302	62.5 (5.9)	1238	55.5 (9.7)	<.001
Maximum coronary artery *z* score, mean (SD)	335	2.2 (3.9)	1400	1.3 (1.5)	<.001
Coronary artery *z* score ≥2	335	84 (25.1)	1400	311 (22.2)	.27
Peak NT-proBNP, median (IQR), ng/L	87	509 (214-1784)	551	4908 (1290-12 900)	<.001
Peak troponin I, median (IQR), ng/L^a^	84	<10 (<10-<10)	732	38 (<10-173)	<.001
Peak creatinine, median (IQR), mg/dL	216	0.33 (0.25-0.45)	1287	0.58 (0.44-0.80)	<.001
Peak CRP, median (IQR), mg/dL	172	8.2 (3.5-15.7)	1205	1.7 (9.9-2.5)	<.001
Peak WBC, median (IQR), /μL	330	15 000 (11 000-21 000)	1269	15 000 (11 000-21 000)	.98
Peak ferritin, median, IQR, ng/L	200	192 (120-330)	1289	501 (277-999)	<.001
Peak d-dimer, median (IQR), μg/mL	141	0.9 (0.5-2.0)	1276	1.9 (1.1-3.0)	<.001
Peak ALT, median (IQR), U/L	190	47 (34-68)	1193	56 (37-91)	.002

Abbreviations: ALT, alanine aminotransferase; CRP, C-reactive protein; ICU, intensive care unit; LVEF, left ventricular ejection fraction; MIS-C, multisystem inflammatory syndrome in children; NT-proBNP, N-terminal pro–brain natriuretic peptide; WBC, white blood cells.

SI conversion factors: To convert ALT to microkatals per liter, multiply by 0.0167; creatinine to micromoles per liter, multiply by 88.4; CRP to milligrams per liter, multiply by 10; d-dimer to nanomoles per liter, multiply by 5.476; ferritin to micrograms per liter, multiply by 1; troponin I to micrograms per liter, multiply by 1; WBC to ×10^9^ per liter, multiply by 0.001.

^a^ Lower bound of detectability was less than 10 and was reported by laboratories as such.

Patients with MIS-C had more severe presentations and worsened clinical outcomes than those with KD (Table 1). Patients with MIS-C were more likely to present with shock and have ICU admission compared with patients with KD. There were no deaths in the KD cohort and 8 deaths in the MIS-C cohort. Patients with MIS-C had lower worst left ventricular ejection fraction (LVEF). Patients with KD had higher peak coronary artery *z* score in any coronary artery branch, but the proportion with any peak coronary artery *z* score greater than or equal to 2 was similar between the 2 patient groups. Patients with MIS-C had higher peak levels of cardiac biomarkers, inflammatory markers, creatinine, and alanine aminotransferase.

**Figure 2.**
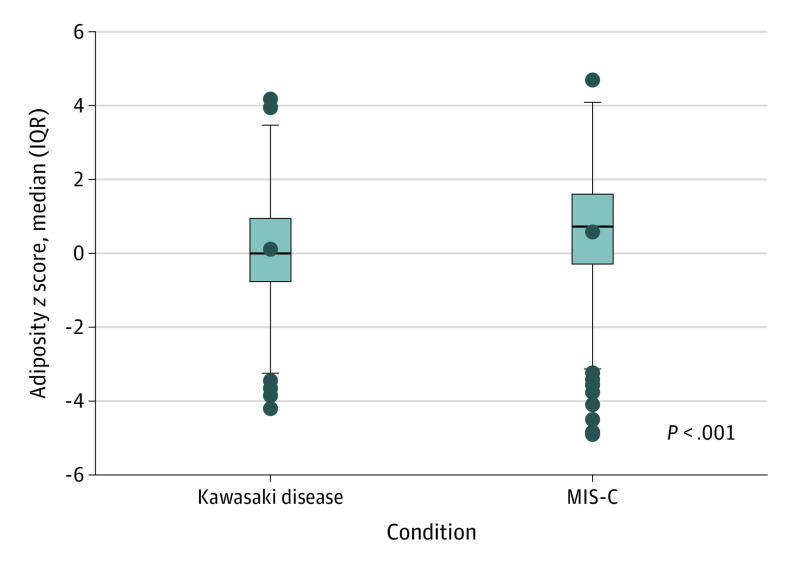
Body Mass Index/Weight *z* Score in Patients With Kawasaki Disease and Multisystem Inflammatory Syndrome in Children (MIS-C) Boxes represent the IQR (first and third quartiles), with the horizontal line within each box representing the median. The horizontal lines at the end of the whiskers represent maximum and minimum values and the dots outside the whiskers represent outlier values.

Patients with MIS-C had significantly higher adiposity *z* scores (*P* < .001) (Figure 2), with the difference remaining significant after adjusting for age, sex, and race and ethnicity (parameter estimate, 0.47 for diagnosis; SE, 0.12; *P* < .001 from general linear modeling). When categorized as adiposity category, patients with MIS-C vs KD had a higher prevalence of both overweight (17.1% vs 11.5%) and obesity (23.7% vs 11.5%) compared with patients with KD (*P* < .001). In addition, patients with MIS-C had a higher prevalence of severe obesity (10.4% vs 3.8%).

**Figure 3.**
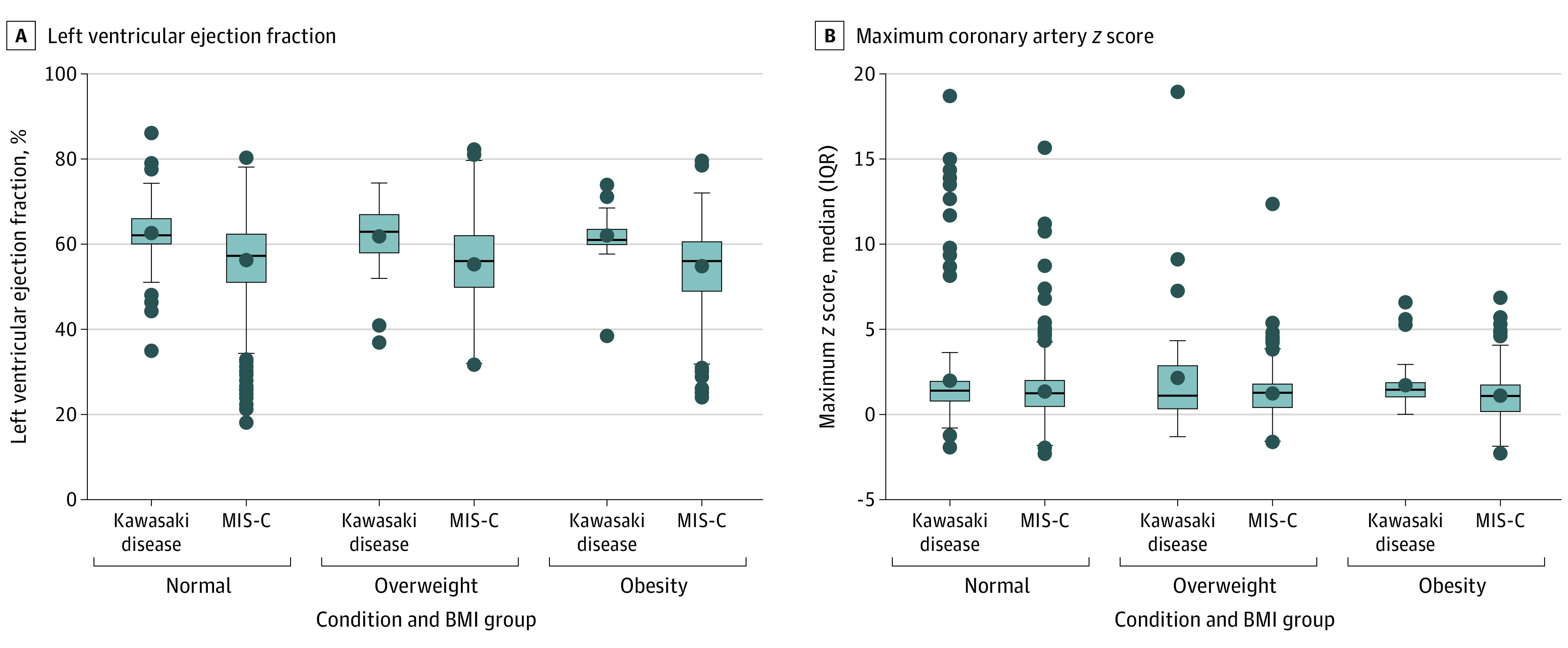
Outcomes in Patients With Kawasaki Disease and Multisystem Inflammatory Syndrome in Children (MIS-C) by Adiposity Category A, Lowest LVEF was significantly lower with increasing adiposity category for patients with MIS-C (*P* < .001) but not KD (*P* = .53). B, Maximum adiposity category was not associated with maximum coronary artery *z* score for patients with KD or MIS-C. Boxes represent the interquartile range (first and third quartiles), with the horizontal line within each box representing the median and the dot within each box representing the mean. The horizontal lines at the end of the whiskers represent maximum and minimum values and the dots outside the whiskers represent outlier values.

Higher adiposity category was not associated with presentation of shock for either KD or MIS-C (Table 2). However, a greater likelihood of ICU admission was observed for higher adiposity categories for both the KD and MIS-C groups. Higher adiposity category was associated with having received inotropic agents only for patients with MIS-C. Lower worst LVEF (Figure 3A) and an LVEF less than 55% were associated with a higher adiposity category for the MIS-C group only. The maximum coronary artery *z* score in any branch (Figure 3B) and *z* score greater than or equal to 2 were not associated with adiposity category for either the KD or MIS-C groups. Higher peak CRP levels, ferritin levels, and white blood cell count, but not d-dimer levels, were associated with a higher adiposity category only for the MIS-C group. Higher peak troponin I but not N-terminal pro–brain natriuretic peptide (NT-proBNP) level was associated with a higher adiposity category for MIS-C only. Higher peak creatinine and alanine aminotransferase levels were both associated with higher adiposity category for MIS-C only.

**Table 2.** Clinical and Laboratory Findings by Adiposity Category in Patients With Kawasaki Disease and Multisystem Inflammatory Syndrome in Children

Characteristic	Kawasaki disease (n = 338)				*P* value	MIS-C (n = 1429)				*P* value
	No.	No. (%)				No.	No. (%)			
		Normal (n = 260)	Overweight (n = 39)	Obesity (n = 39)			Normal (n = 846)	Overweight (n = 244)	Obesity (n = 338)	
Shock at presentation	332	5/254 (2.0)	3/39 (7.7)	1/39 (2.6)	.37	1426	274/844 (32.5)	90/244 (36.9)	123/338 (36.4)	.15
ICU admission	335	10/257 (3.9)	5/39 (12.8)	7/39 (18.0)	<.001	1428	463/846 (54.7)	143/244 (58.6)	209/338 (61.8)	.03
Received inotropic agents	338	19/260 (7.3)	5/39 (12.8)	3/39 (7.7)	.65	1428	363/846 (42.9)	110/244 (45.1)	175/338 (51.8)	.007
Hospital length of stay, median (IQR), d	338	5 (4-6) [n = 260]	5 (4-7) [n = 39]	5 (4-8) [n = 39]	.48	1429	7 (5-10) [n = 846]	8 (5-11) [n = 244]	7 (5-11) [n = 339]	.003
Worst LVEF, mean (SD), %	302	62.7 (5.6) [n = 231]	61.6 (7.8) [n = 35]	62.0 (5.7) [n = 36]	.53	1238	56.3 (9.6) [n = 726]	55.6 (9.5) [n = 214]	53.8 (9.7) [n = 298]	<.001
LV systolic dysfunction (LVEF<55%)	302	11/231 (4.8)	3/35 (8.6)	1/36 (2.8)	.91	1238	250/725 (34.4)	92/214 (43.0)	140/298 (47.0)	<.001
Maximum coronary artery *z* score, mean (SD)	335	2.3 (4.2) [n = 257]	2.1 (3.4) [n = 39]	1.7 (1.4) [n = 39]	.65	1400	1.4 (1.4) [n = 827]	1.3 (1.4) [n = 238]	1.2 (1.8) [n = 335]	.92
Coronary artery *z* score ≥2	335	62/257 (24.1)	14/39 (35.9)	8/39 (20.5)	.91	1400	200/827 (24.2)	44/238 (18.5)	67/335 (20.0)	.07
Peak CRP, median (IQR), mg/dL	172	86.5 (39.9-155.2) [n = 132]	73.0 (17.1-183.2) [n = 23]	73.0 (28.1-178.9) [n = 17]	.93	1205	162.6 (84.8-236.1) [n = 713]	179.1 (110.7-253.0) [n = 206]	205.6 (135.7-302.0) [n = 286]	<.001
Peak NT-proBNP, median (IQR), ng/L	87	482.8 (200.0-1239.0) [n = 60]	1783.9 (209.0-2536.2) [n = 17]	427.5 (311.6-3054.0) [n = 10]	.42	551	4963.5 (1383.0-12 800.0) [n = 334]	3520.0 (926.0-12 491.9) [n = 89]	5133.5 (1076.0-14 900.0) [n = 128]	.81
Peak troponin I, median (IQR), ng/L^a^	84	<10 (<10-<10) [n = 58]	<10 (<10-77) [n = 17]	<10 (<10 -<10) [n = 9]	.10	714	30 (<10-123) [n = 434]	36 (<10-142) [n = 127]	60 (10-287) [n = 171]	.03
Peak ALT, median (IQR), U/L	190	47 (33-68) [n = 145]	44 (35-55) [n = 20]	52 (35-106) [n = 25]	.62	1193	56 (37-90) [n = 707]	50 (35-77) [n = 195]	61 (39-109) [n = 291]	.02
Peak creatinine, median (IQR), mg/dL	216	0.31 (0.25-.44) [n = 164]	0.38 (0.23-0.51) [n = 25]	36 (54-46) [n = 27]	.05	1287	0.54 (0.40-0.74) [n = 755]	0.60 (0.48-0.84) [n = 219]	0.66 (0.50-0.95) [n = 313]	<.001
Peak WBC, median (IQR), /μL	330	15 000 (11000-21 000) [n = 257]	14 000 (11 000-19 000) [n = 37]	16 000(11 000-22 000) [n = 36]	.83	1269	15 000 (10 000-20 000) [n = 757]	17 000 (11 000 – 24 000) [n = 222]	16 000 (11 000-23 000) [n = 290]	.002
Peak ferritin, median (IQR), ng/L	200	187 (109-308) [n = 148]	232 (119-429) [n = 27]	179 (109-766) [n = 25]	.28	1289	471 (262-924) [n = 755]	521 (260-1179) [n = 219]	595 (317-1215) [n = 315]	.005
Peak d-dimer, median (IQR), μg/mL	141	0.9 (0.5-1.9) [n = 102]	0.9 (0.6-2.2) [n = 22]	0.5 (0.4-2.0) [n = 17]	.89	1276	1.8 (1.1-3.0) [n = 754]	1.9 (1.1-3.1) [n = 219]	1.9 (1.2-3.1) [n = 303]	.80

Abbreviations: ALT, alanine aminotransferase; CRP, C-reactive protein; ICU, intensive care unit; LV, left ventricular; LVEF, left ventricular ejection fraction; MIS-C, multisystem inflammatory syndrome in children; NT-proBNP, N-terminal pro-brain natriuretic peptide; WBC, white blood cells.

SI conversion factors: To convert ALT to microkatals per liter, multiply by 0.0167; creatinine to micromoles per liter, multiply by 88.4; CRP to milligrams per liter, multiply by 10; d-dimer to nanomoles per liter, multiply by 5.476; ferritin to micrograms per liter, multiply by 1; troponin I to micrograms per liter, multiply by 1; WBC to ×10^9^ per liter, multiply by 0.001.

^a^ Lower bound of detectability was less than 10 and was reported by laboratories as such.

## Discussion

While there is an overlap between the clinical presentation of KD and MIS-C, distinct differences exist with respect to their predilection for clinical outcomes. Moreover, the comorbidities and risk factors that may be associated with important clinical outcomes may be different between the 2 disease processes. Risk stratification in KD largely concerns the potential for coronary artery aneurysm development with known risk factors, such as the presence of coronary artery involvement at presentation, young age (<12 months), and resistance to intravenous immunoglobulin treatment.^3,26^ Obesity, to date, has been understudied as a potential risk factor for children who develop KD. Conversely, obesity, among other comorbid conditions, has been explored when evaluating the development and severity of MIS-C.^27,28^ Given that the excessive dysregulated adipose tissue present in individuals with obesity appears to affect a systemic inflammatory state,^19^ it is possible that obesity may mediate disease severity in both KD and MIS-C. In the present study, obesity was significantly more prevalent in patients with MIS-C than KD (23.7% vs 11.5%). While obesity was associated with ICU admission in patients with KD, it was not associated with other important clinical outcomes, including coronary artery involvement. For MIS-C, obesity was associated with important clinical outcomes, including ICU admission, longer hospital length of stay, worse LVEF, worsened inflammatory markers, and increased troponin I, liver enzyme, and creatinine levels. These findings further highlight that KD and MIS-C, while similar in many ways, represent 2 distinct inflammatory processes with unique risk factors and varying outcomes.

The prevalence of obesity among patients with MIS-C in the present study (23.7%) is quite similar to prevalence values identified in both the general population^29^ and in other studies of children with MIS-C.^30,31,32,33^ In a study of MIS-C cases reported to the Centers for Disease Control and Prevention in 2020 and 2021, 25% had obesity.^33^ Similarly, in a systematic review and meta-analysis of patients with MIS-C, obesity was present in 25% of the children.^30^ The lower prevalence of obesity observed in patients with KD in our study may reflect known lower rates of obesity in younger compared with older children,^29^ as patients with KD were significantly younger than those with MIS-C. Differences in the prevalence of obesity related to race and ethnicity may also be a confounding factor. However, the adiposity *z* score remained higher in the present study for patients with MIS-C after adjusting for age, sex, and race and ethnicity. With respect to KD, the prevalence of overweight and obesity (both 11.5%) was similar to a previous study of Chinese children with KD (18.5%).^18^

Concerning clinical outcomes, patients with MIS-C in the present study had more severe clinical presentations and disease courses than those with KD. Whereas there were no deaths among patients with KD, there were 8 deaths in those with MIS-C. Patients with MIS-C also had a substantially higher prevalence of cardiogenic shock at presentation, ICU admission, and worse left ventricular systolic function. Patients with KD, however, had a higher mean maximal coronary artery *z* score than those with MIS-C. These findings are similar to earlier work from our group of investigators that compared SARS-CoV-2–positive patients with KD (presumed MIS-C with a KD-like presentation) with confirmed SARS-CoV-2–negative patients with KD.^21^ Moreover, the severity of presentation, multiorgan involvement, and need for ICU care for patients with MIS-C has been reported by other investigators.^2,5^

In the present study, in patients with KD, obesity was not associated with clinical outcomes apart from ICU admission rates. Specifically, obesity was not associated with the maximum coronary artery *z* score or the presence of coronary artery lesions. This is in contrast to an earlier study that evaluated obesity in patients with KD, where the presence of overweight and obesity was associated with increased odds of coronary artery aneurysms.^18^ However, in this prior study, patients with overweight or obesity more commonly received nonstandard intravenous immunoglobulin treatment (eg, dosing split over 2 days), thus potentially confounding the results. The rationale for the higher ICU admission in our study among patients with KD remains unclear, particularly as other related clinical measures (eg, shock at presentation and left ventricular systolic function) were not significantly different for patients with obesity.

Prior population-based studies have reported that obesity is associated with the development or presence of MIS-C among children hospitalized with a COVID-19 illness.^10,12,27^ Other work, however, has observed a similar prevalence of obesity among children hospitalized with MIS-C (36.2%) and those hospitalized with severe acute COVID-19 infections (41.8%).^5^ Associations between obesity and the severity of presentation and occurrence of adverse outcomes in children with MIS-C have similarly yielded mixed results.^6,12,15,16,17^For example, in a Polish study of 306 children with MIS-C, obesity was not associated with the duration of symptoms, length of hospital stay (including ICU stay), frequency of cardiovascular abnormalities, or differences in laboratory markers, such as peak CRP, NT-proBNP, ferritin, and serum creatinine levels, or white blood cell count.^15^ Similarly, in a retrospective registry study, no significant differences were identified regarding critical illness, ICU admission, or mechanical ventilation rates in patients with MIS-C with obesity compared with those without.^12^ However, obesity was associated with a longer hospital length of stay. In a multicenter retrospective surveillance study involving 1080 patients with MIS-C, obesity was not associated with ICU admission, but was associated with decreased cardiac function.^16^ Conversely, other work that has evaluated children hospitalized with COVID-19 infections (including those hospitalized with MIS-C) have identified obesity as an independent risk factor for more severe disease.^6,17^ The present study, however, did not differentiate the associations between obesity and MIS-C outcomes from associations between obesity and acute COVID-19 infection outcomes. We have built on previous work in the present study by comprehensively analyzing the associations between obesity and numerous critical clinical outcomes in a large international cohort of patients with MIS-C. Apart from coronary artery lesions, obesity was associated with worse clinical presentations and adverse outcomes, including the use of inotropes, adverse cardiac and kidney parameters, and worsened inflammatory markers.

### Strengths and Limitations

Through the multi-institutional IKDR, we evaluated worldwide data on patients with MIS-C and KD, allowing for a detailed analysis with a large sample size evaluating the associations between obesity and both disease processes. However, some limitations should be considered when interpreting the study results. First, we excluded patients who met the criteria for KD but had either an unknown or positive COVID-19 infectious status. This stringent criterion may have excluded some true KD cases; however, this approach was necessary to avoid misclassification, given the clinical overlap between KD and MIS-C. To this end, earlier work from the IKDR has reported that COVID-positive patients with KD had a tendency toward a demographic and clinical profile suggestive of MIS-C.^21^ As a result, 499 potential patients with KD were excluded from the analysis, thus potentially limiting the power of our analysis. Moreover, several variables had missing data, particularly for patients with KD, which is likely because these variables are not commonly collected in the clinical setting (eg, NT-proBNP and troponin I). Data pertaining to race and ethnicity, which is associated with obesity and was included in our regression modeling, was missing for 31% of the study population. As BMI is not conventionally used in children younger than 2 years,^29^ a weight *z* score was used for children younger than 2 years (primarily with KD). This should be considered when interpreting prevalence values for adiposity categories. Some variables are naturally increased in individuals with obesity and may not reflect more severe disease. For example, alanine aminotransferase levels are known to be increased in those with obesity,^34^ and thus may not have reflected true end-organ injury in patients with MIS-C. Data regarding socioeconomic status were not available. While this was an international cohort study, most patients were from the US or Canada. In addition, although detailed data quality checks are in place at the DCC, this study remains vulnerable to the limitations inherent to registry-based analyses, including human error and variations in laboratory and testing assessment accuracy.

## Conclusions

In this multi-institutional cohort study from the IKDR, obesity was more prevalent in patients with MIS-C compared with children with KD. For children with MIS-C, obesity was associated with a more severe clinical presentation and, of course, higher levels of inflammatory markers; cardiac, kidney, and hepatic dysfunction; and worsened cardiac biomarkers. Obesity status was largely not associated with worsened outcomes in patients with KD, apart from a greater need for ICU admission. The differing prevalence of obesity and associations with disease severity between KD and MIS-C support the evidence that, while substantial overlap exists, KD and MIS-C represent 2 distinct inflammatory disease processes. These study findings suggest that obesity as a comorbid factor should be considered at clinical presentation in children with MIS-C.

## Notes

Supplement 1. **Nonauthor Collaborators.** The International Kawasaki Disease Registry

Supplement 2.
Data Sharing Statement
