# Supplementary material for: Obesity and Outcomes of Kawasaki Disease and COVID-19–Related Multisystem Inflammatory Syndrome in Children
Source: JAMA Netw Open. 2023 Dec 8;6(12):e2346829. doi: 10.1001/jamanetworkopen.2023.46829 (PMC10709775; doi:10.1001/jamanetworkopen.2023.46829)
Supplement: Supplement 1. — Nonauthor Collaborators. The International Kawasaki Disease Registry [file jamanetwopen-e2346829-s001.pdf]

Supplemental Online Content: Nonauthor Collaborators

*Indicates required information. Only first name, last name, and suffix will appear in PubMed.

***Group Name(s): International Kawasaki Disease Registry**

***First Name and Middle Initial(s)**	***Last Name**	***Suffix (eg, Jr, III)**	Academic Degrees	Institution	Location (city, state/province, country)	Role or Contribution, eg, chair, principal investigator	Group (if more than 1 Group listed in the byline) and/or Subgroup (eg, Steering Committee)
Mahmoud	Alsalehi		MD	Kingston Health Science Centre, Kingston, ON, Canada	Kingston, ON, Canada	Site PI	
Jean A.	Ballweg		MD	Children's Hospital and Medical Center. University of I	Omaha, NE, USA	Site CO-PI	
Benjamin T.	Barnes		MD	Johns Hopkins School of Medicine, Baltimore MD, USA	Baltimore MD, USA	Site PI	
Elizabeth	Braunlin		MD, PHD	University of Minnesota, Minneapolis, MN, USA	Minneapolis, MN, USA	Site PI	
Ashley	Buffone		MD	Division of Pediatric Cardiology, CHU Ste-Justine, Univ	Montreal, QC, Canada	Site Study Coordinator	
Juan Carlos	Bustamante-Ogando		MD	Hospital Angeles Lomas, Huixquilucan, Mexico	Huixquilucan, Mexico	Site Study Coordinator	
Arthur J.	Chang		MD	Jacobs School of Medicine and BioMedical Sciences, U	Buffalo, NY, USA	Site CO-PI	
Paul	Dancey		MD, FRCPC	Janeway Children's Health and Rehabilitation Centre,	Saint John's, NL, Canada	Site PI	
Sarah D.	De Ferranti		MD, MPH	Boston Children's Hospital, Harvard Medical School, B	Boston, MA, USA	Site CO-PI	
Catherine	Dimes		RN	The Heart Center at Nationwide Children's Hospital, C	Columbus, OH, USA	Site Study Coordinator	
Audrey	Dionne		MD	Boston Children's Hospital, Harvard Medical School, B	Boston, MA, USA	Site PI	
Mona	El Ganzoury		MD, PhD	Pediatric Cardiology Division, Department of Pediatric	Cairo, Egypt	Site CO-PI	
Nora	El Samman		MD, PhD	Pediatric Cardiology Division, Department of Pediatric	Cairo, Egypt	Site CO-PI	
Elisa	Fernández Cooke		MD, PhD	Hospital 12 de Octubre (Imas12), Madrid, Spain	Madrid, Spain	Site PI	
Luis Martin	Garrido-Garcia		MD, MSc	Hospital Angeles Lomas, Huixquilucan, Mexico and Ins	Mexico City, Mexico	Site PI	
Therese M.	Giglia		MD	Children's Hospital of Philadelphia, Philadelphia, PA, U	Philadelphia, PA, USA	Site CO-PI	
Guillermo Larios	Goldenberg		MD	Hospital Clínico Red de Salud UC-Christus, Pontificia U	Santiago, Chile	Site PI	
Kevin C.	Harris		MD, MHSc	Children's Heart Centre, BC Children's Hospital and Ur	Vancouver, BC, Canada.	Site PI	
Mark D.	Hicar		MD, PhD	Jacobs School of Medicine and BioMedical Sciences, U	Buffalo, NY, USA	Site PI	
Nicolas M.	Hidalgo Corral		MD	Childrens Hospital at Montefiore, Albert Einstein Colle	Bronx, NY, USA	Site CO-PI	
Supriya S.	Jain		MD	New York Medical College/Maria Fareri Children's Hos	Valhalla, NY, USA	Site PI	
Pei-Ni	Jone		MD	Children's Hospital Colorado, University of Colorado A	Aurora, CO, USA	Site PI	
Hidemi	Kajimoto		MD, PhD	Seattle Children's Research Institute, Seattle, WA, USA	Seattle, WA, USA	Site Study Coordinator	
Shelby	Kutty		MD, PhD	Johns Hopkins School of Medicine, Baltimore MD, USA	Baltimore MD, USA	Site CO-PI	
Marcello	Lanari		MD, PhD	Pediatric Emergency Unit, IRCCS Azienda Ospedaliero	Bologna, Italy	Site Study Coordinator	
Robert W.	Lowndes		.	Children's National Hospital, The George Washington	Washington, DC, USA	Site Study Coordinator	
Victoria	Maksymiuk		BSc	New York Medical College/Maria Fareri Children's Hos	Valhalla, NY, USA	Site Study Coordinator	
Daniel	Mauriello		MD	Johns Hopkins All Children's Hospital, Saint Petersburg	Saint Petersburg, FL, USA	Site PI	
Kimberly E.	McHugh		MD, MScR	Medical University of South Carolina, Charleston, SC, I	Charleston, SC, USA	Site PI	
Shae A.	Merves		MD	Arkansas Children's Hospital/University of Arkansas fo	Little Rock, AR, USA	Site PI	
Nilanjana	Misra		MBBS, FAAP, FSCMR	Cohen Children's Medical Center, Northwell Health, Q	Queens, NY, USA	Site PI	
Sindhu	Mohandas		MD	Children's Hospital of Los Angeles, Los Angeles, CA, US	Los Angeles, CA, USA	Site Study Coordinator	
Tapas	Mondal		MD	Division of Cardiology, Department of Pediatrics, McM	Hamilton, ON, Canada	Site PI	
Todd T.	Nowlen		MD	Phoenix Children's Hospital, Phoenix, AZ, USA	Phoenix, AZ, USA	Site PI	
Deepa	Prasad		MD	Banner Children's at Desert Medical Center, Mesa, AZ	Mesa, AZ, USA	Site PI	
Prasad	Ravi		MD	Banner Children's at Desert Medical Center, Mesa, AZ	Mesa, AZ, USA	Site CO-PI	
Arash A.	Sabati		MD	Phoenix Children's Hospital, Phoenix, AZ, USA	Phoenix, AZ, USA	Site CO-PI	
Anupam	Sehgal		MBBS	Kingston Health Science Centre, Kingston, ON, Canada	Kingston, ON, Canada	Site CO-PI	
Ashish	Shah		MD, MBA	Johns Hopkins All Children's Hospital, Saint Petersburg	Saint Petersburg, FL, USA	Site CO-PI	

Supplemental Online Content: Nonauthor Collaborators

*Indicates required information. Only first name, last name, and suffix will appear in PubMed.

***First Name and Middle Initial(s)**	***Last Name**	***Suffix (eg, Jr, III)**	Academic Degrees	Institution	Location (city, state/province, country)	Role or Contribution, eg, chair, principal investigator	Group (if more than 1 Group listed in the byline) and/or Subgroup (eg, Steering Committee)
Balasubramanian	Sundaram		MD	Kanchi Kamakoti Childs Trust Hospital, Chennai, India	Chennai, India	Site CO-PI	
Belén	Toral Vázquez		MD	Hospital 12 de Octubre (Imas12), Madrid, Spain	Madrid, Spain	Site Study Coordinator	
Adriana H.	Tremoulet		MD	University of California San Diego/Rady Children’s Hos	San Diego, CA, USA	Site CO-PI	
Aishwarya	Venkataraman		MRCPCH	Kanchi Kamakoti Childs Trust Hospital, Chennai, India	Chennai, India	Site CO-PI	
Marco Antonio	Yamazaki-Naksahimada		MD	Instituto Nacional de Pediatría, Mexico City, Mexico	Mexico City, Mexico	Site CO-PI	
Anji T.	Yetman		MD	Children's Hospital and Medical Center. University of I	Omaha, NE, USA	Site PI	
Varsha	Zadokar		MBBS	Nemours Children's Hospital, Wilmington, DE, USA	Wilmington, DE, USA	Site Study Coordinator	
