# Supplementary material for: Obesity and Outcomes of Kawasaki Disease and COVID-19–Related Multisystem Inflammatory Syndrome in Children
Source: JAMA Netw Open. 2023 Dec 8;6(12):e2346829. doi: 10.1001/jamanetworkopen.2023.46829 (PMC10709775; doi:10.1001/jamanetworkopen.2023.46829)
Supplement: Supplement 2. — Data Sharing Statement [file jamanetwopen-e2346829-s002.pdf]

Data Sharing Statement

Khoury. Obesity and Outcomes of Kawasaki Disease and COVID-19–Related Multisystem Inflammatory Syndrome in Children. *JAMA Netw Open*. Published December 08, 2023. doi:10.1001/jamanetworkopen.2023.46829

Data
Data available: No

Additional Information
Explanation for why data not available: Individual REB stipulations of different centers within the IKDR prevent data sharing outside the IKDR
